# MultiLink Analysis: Brain Network Comparison via Sparse Connectivity Analysis

**DOI:** 10.1038/s41598-018-37300-4

**Published:** 2019-01-11

**Authors:** Alessandro Crimi, Luca Giancardo, Fabio Sambataro, Alessandro Gozzi, Vittorio Murino, Diego Sona

**Affiliations:** 1 Pattern Analysis and Computer Vision, Istituto Italiano di Tecnologia, Genova, Italy; 2 Center for Precision Health, School of Biomedical Informatics, University of Texas Health Science Center at Houston, Houston, USA; 3 Department of Experimental and Clinical Medical Sciences, University of Udine, Udine, Italy; 4 Functional Neuroimaging Laboratory, Istituto Italiano di Tecnologia, Rovereto, Italy; 5 Department of Computer Science, University of Verona, Verona, Italy; 6 Neuroinformatics Laboratory, Fondazione Bruno Kessler, Trento, Italy; 7 Institute of Neuropathology, University Hospital of Zürich, Zürich, Switzerland

## Abstract

The analysis of the brain from a connectivity perspective is revealing novel insights into brain structure and function. Discovery is, however, hindered by the lack of prior knowledge used to make hypotheses. Additionally, exploratory data analysis is made complex by the high dimensionality of data. Indeed, to assess the effect of pathological states on brain networks, neuroscientists are often required to evaluate experimental effects in case-control studies, with hundreds of thousands of connections. In this paper, we propose an approach to identify the multivariate relationships in brain connections that characterize two distinct groups, hence permitting the investigators to immediately discover the subnetworks that contain information about the differences between experimental groups. In particular, we are interested in data discovery related to connectomics, where the connections that characterize differences between two groups of subjects are found. Nevertheless, those connections do not necessarily maximize the accuracy in classification since this does not guarantee reliable interpretation of specific differences between groups. In practice, our method exploits recent machine learning techniques employing sparsity to deal with weighted networks describing the whole-brain macro connectivity. We evaluated our technique on functional and structural connectomes from human and murine brain data. In our experiments, we automatically identified disease-relevant connections in datasets with supervised and unsupervised anatomy-driven parcellation approaches and by using high-dimensional datasets.

## Introduction

The analysis of brain networks, or connectomes, is a recent and exciting advancement in magnetic resonance imaging (MRI) that promises to identify new phenotypes for healthy, diseased or aging brains^1^. A connectome is a comprehensive map of the connections in the brain, which is conceived as a network, where brain areas (nodes) are connected by links (edges)^2^, and connections can be either given by white matter tracts between pairs of brain regions or by an index of the correlation of functional activity^3^. This approach allows for analyzing the brain as a complex system of dynamically interacting components without explicitly relying on local activation or brain morphology.

### Case-control studies and connectomics

Experiments with connectomes are typically designed by comparing a studied group with a control group to identify brain-network topological biomarkers relevant to the studied group^4^. Indeed, intergroup differences in some of these topological measures have been discovered for various neuropsychiatric disorders^5^, such as Alzheimer’s disease^6^, multiple sclerosis^7^, schizophrenia^8^, stroke^9^, major depression^10^, and autism spectrum disorder^11^. All these approaches use topological measures with statistical tests to assess their discrimination power in a univariate analysis framework. Alternatively, in a multivariate framework, machine learning methods have been proposed to differentiate groups of subjects using topological measures^12^. Surveys on graph-topological metrics using functional magnetic resonance imaging (fMRI) data and related clinical applications using structural features have been presented in Varoquaux *et al*.^13^ and Griffa *et al*.^14^, respectively.

### Local differences between connectomes

The main drawback of the aforementioned approaches is the limited interpretability of the graph statistics, as they miss the local characterization of the groups in terms of the differences in the connectivity; instead, they employ global statistics that are difficult to translate into clinical settings for local analysis. A method that allows insights on local connectivity patterns in case-control studies relies on network-based statistics (NBS). In this approach, the connectivity between pairs of brain regions is tested for significance using univariate statistics for functional^15^ and anatomical^16^ connectivity disturbances. Simpson *et al*.^17^ extended the NBS method using a permutation test based on the Jaccard index at the node level, while Chen *et al*.^18^ enhanced NBS by regulating the topological structures. Other research groups^19–21^ leveraged support vector machine (SVM) weights to identify discriminating regions. SVM is a supervised learning method that constructs a hyperplane or set of hyperplanes in a high- or infinite-dimensional space used for classification. Specifically, Ng *et al*.^20^ projected covariance estimates onto a common tangent space to reduce the statistical dependencies between elements. Then, while Mastrovito *et al*.^19^ employed recursive feature elimination (RFE) to identify connections relevant for the classification, both Goankar *et al*.^21^ and Ng *et al*.^20^ found meaningful connections using t-tests on the SVM weights. A more advanced machine learning approach is based on SVM coupled with Riemannian/Grassmannian geometry^22^. Van Heuvel *et al*.^23^ proposed a subgraph level analysis for more specific and localized information, with a specific emphasis on the potential functional importance of highly connected hubs (“rich-clubs”). Although the focus on rich-clubs is insightful, this method can leave out subtle differences between case-control groups that are not present in highly connected hubs. Finally, despite NBS and its extensions outperform other methods in comprehensive comparisons, the identification of graph subnetworks is a prerequisite that can limit the detected connections, and the t-tests are carried out in a univariate manner^24^. Moreover, the choice of related statistics can considerably influence the results^24^.

### Relation to previous methods

In this context, we are interested in data discovery related to connectomics, where the connections that characterize differences between two groups of subjects are found, and where maximizing accuracy does not guarantee reliable interpretation since similar accuracies can be obtained from distinct sets of features^25^. To overcome the limitations of the univariate approaches, which perform statistical tests on single connections as mentioned in the previous subsection—and in particular to the most commonly used NBS^26^—we use a multivariate bootstrap-like approach followed by a stability selection step. Therefore, we propose a fully data-driven method to identify relevant brain subnetworks in experiments with a case-control design that can be used as a hypothesis generation tool for connectome investigations. Our method has the potential to work equally well with functional and structural MRI data, and no prior knowledge about the type of connectivity is required—only examples of brain connectivity matrices of two groups are needed.

A similar method proposed by McMenamin and Pessoa^27^ implemented a two-layer dimensionality reduction technique based on principal component analysis (PCA), followed by quadratic discriminant analysis to identify clusters with altered connectivity at the voxel level. However, when PCA was used for feature selection, the eigenvalues of the covariance matrix were used regardless of the prior knowledge of the groups to be discriminated, and in doing so, the resulting features may not be those that were truly meaningful in terms of discrimination between groups. Conversely, our method directly performs a sparse version of linear discriminant analysis (LDA) that, by design, tries to optimize the feature selection step aimed at discriminating the groups. This approach allows the proposed method to be more specific in terms of identified discriminating connections. Furthermore, sparse models follow a feature selection agenda to subselect among existing variables, whereas PCA dimensionality reduction follows a feature engineering agenda to generate a set of new variables. Feature selection by a sparse model is similar to the RFE used by Mastrovito *et al*.^19^. However, the stability of the RFE approach depends heavily on the type of model used for feature ranking at each iteration, and as shown empirically, using regularized ridge regression jointly with stability selection criteria can provide more stable results in terms of the *stability selection* of features and yields finite sample familywise error control^28,29^. More specifically, the proposed model is based on an ensemble of sparse linear discriminant models allowing to find the networks’ elements (a set of edges) that can consistently distinguish two groups, in the attempt to minimize the subset of selected connectivity features and simultaneously maximize the difference between the groups^30^. Essentially, the system acts as a *filter* that removes the elements that are not useful for discriminating between the groups. First, the system enforces sparsity at the individual level. Then, the system performs the second stage of feature filtering across the dataset to assure stability selection. This feature selection process is not inherently specific to connectomes, as it can be applied to arbitrary high-dimensional, multivariate datasets. Nevertheless, recent studies have shown that a sparsity-based approach can be particularly useful in graph/connectome analysis, as they can highlight significant connections when prior knowledge is missing^24,31–33^.

Other methods have used sparsity to estimate relevant connections^34–36^. However, these methods did not focus on finding the discriminant connections between groups while performing sparse selection. These methods use sparsity to reduce the number of connections regardless of interclass discrimination.

#### MultiLink Analysis (MLA)

The interpretation of differences in brain networks is not always straightforward given the individual variability and the high dimensionality of data^37^. Moreover, the internal structure of the brain connectivity with cross-relationships and dependencies in the feature space (the edges) may prevent a full retrieval of the groups’ differences using univariate analysis. Machine learning and dimensionality reduction techniques are designed to solve these issues, and hence, these methods are a natural choice for addressing this discrimination task. We propose a two-stage feature selection process. In the first stage, a classifier reinforcing sparsity is employed to select the discriminant features, iterating over different subsamples of the dataset in a bootstrapping framework. Then, only features that are consistently selected across the iterations are retained according to a stability selection criterion.

An approach that simultaneously implements classification and feature selection in a sparse framework is *sparse logistic regression*, which has been used to select relevant voxels for decoding fMRI activity patterns^38,39^. Alternatively, in the case of Gaussian-distributed data, the well-known *linear discriminant analysis* has been extended to the sparse case with the *sparse discriminant analysis* (SDA) model^30,40^. In particular, the method by Clemmensen *et al*.^30^ implements the elastic net regression with the ${\ell }_{1}$-norm on the feature weights that indirectly sets the number of selected features.

For all the experiments, the connectivity matrices are vectorized and ordered as rows in an *n* × *p* data-matrix **X**, where *n* is the number of observations and *p* is their dimensionality. The corresponding classification of objects is encoded into the *n* × *K* indicator matrix **Y**, where each cell **Y**_*ik*_ indicates whether observation *i* belongs to class *k*. The SDA proposed by^30^ then finds the discriminant vectors *β*_*k*_ for each class *k* and the vector of scores *θ*_*k*_ by the convex optimization given by the following regularized linear discriminant formulation:

1 $$\{\begin{array}{l}{{\rm{\min }}}_{{\beta }_{k},{\theta }_{k}}\,\parallel {\bf{Y}}{\theta }_{k}-{\bf{X}}{\beta }_{k}{\parallel }^{2}+\eta \parallel {\beta }_{k}{\parallel }_{1}+\gamma \,{\beta }_{k}^{T}{\rm{\Omega }}\,{\beta }_{k},\\ \begin{array}{ll}{\rm{subject}}\,{\rm{to}} & \tfrac{1}{n}{\theta }_{k}^{T}{{\bf{Y}}}^{T}{\bf{Y}}{\theta }_{k}=1,\\  & {\theta }_{k}^{T}{{\bf{Y}}}^{T}{\bf{Y}}{\theta }_{l}=0\forall l < k.\end{array}\end{array}$$

where Ω is an arbitrary positive definite matrix, which allows calculating smooth discriminant vectors *β*_*k*_ even if the number of samples is smaller than the number of features ($n\ll p$). In our experiments, we used Ω = **I**, which makes the formulation an elastic net problem (${\beta }_{k}^{T}\,{\rm{\Omega }}\,{\beta }_{k}=\parallel {\beta }_{k}{\parallel }_{2}$). The nonnegative parameters *η* and *γ* control the ${\ell }_{1}$ and ${\ell }_{2}$ regularizations, respectively. The parameter *η* can also be reformulated as the number of desired variables that are left in the model, and when used in this context, we refer to it as *α*^41^.

The advantage of the proposed sparse method is its capability of managing high-dimensional data due to the ${\ell }_{2}$ regularization. Moreover, the ${\ell }_{1}$ regularization term allows the model to select a small subset of features for linear discrimination. This term might result in a loss of predictive power but a reduction in the overfitting problem. In contrast, the ${\ell }_{2}$ penalty term has the grouping effect property, i.e., it maintains small and comparable weights of the correlated predictors^41^. Moreover, the ${\ell }_{2}$ penalty term is much better at minimizing the prediction error than ${\ell }_{1}$ regularization. As a result, their combination allows the determination of a good trade-off between an optimal classifier and a minimal selection of relevant predictors. Further details on the regularization parameters are given in the Method section and the Appendix.

### Experimental overview

Owing to the sparsity principle driving the learning method combined with the statistical robustness of ensemble methods, our multivariate approach can scale up with the number of analyzed connections, even when employing a limited number of whole-brain connectivity matrices. By virtue of being multivariate, this approach can identify brain subnetworks whose edges, when considered as a set, can characterize the differences between the connectomes but cannot when considered independently. Moreover, the method does not have to rely on covariance matrices but only requires an index describing the strength of the connectivity between the areas in terms of correlation, similarity, dissimilarity or other metrics. For example, in the case of structural connectivity, the matrix can be determined by counting the number of connections between the areas.

We validated the approach on three real datasets. In the first experiment, we used the structural connectivity based on the tractography extracted from diffusion tensor imaging (DTI). Specifically, we compared a group of acallosal BTBR mice (a well-characterized model of autism) with a group of control normocallosal and normosocial C57BL/6J mice^42,43^. Performing this experiment with a simple and well-known connectivity dysfunction, without the use of any prior anatomical parcellation to avoid any prior bias, we empirically validated the approach, which retrieved the expected dissimilarity between the two groups.

A further experiment was conducted on structural connectivity matrices from a publicly available dataset of patients affected by Alzheimer’s disease, where connectivity was also defined by tractography. The final experiment was carried out on a large functional dataset of attention deficit hyperactivity disorder (ADHD) children compared to typically developing (TD) children. Further details are given in the Method section.

In all cases, our method successfully detected intergroup differences relevant to the medical condition investigated. Those results were compared to the results obtained by using NBS, and a framework based on SVM weights^20,21^. NBS and MLA select discriminative features in different ways. NBS performs univariate t-tests among the features, while MLA performs a sparse multivariate regression. Nevertheless, NBS is the commonly used algorithm for this type of analysis and is considered the state of the art. The SVM-based method is a machine learning approach that we used to investigate the most significant connections obtained from the SVM discrimination weights, similar to previous studies^20,21^. Selected weights are those larger than the 95-th percentile or smaller than the 5-th percentile of a random weight distribution representing the null hypothesis. The null hypothesis for the SVM weights is obtained by performing 1000 random permutations of the labels of the two groups. In our experiments, we used the LibSVM toolbox^44^.

All experiments were conducted in accordance with relevant guidelines and regulations. The human experiments used publicly available datasets. The Alzheimer experiments were conducted on data previously acquired by the ADNI initiative according to good clinical practice guidelines, US 21 CFR Part 50 – Protection of Human Subjects, and Part 56 – Institutional Review Boards, acquiring both phone and written consent. The data are from different centers, though the umbrella Institutional Review Board that approved the study and protocol was the University of California, San Francisco. The ADHD experiments were also conducted on data previously acquired for another study, for which the ethics review board of New York University granted the ethical approval and for which informed consent was obtained for each subject. The murine experiments were conducted in accordance with Italian law (DL 116, 1992 Ministero della Sanitá, Roma) and the recommendations in the Guide for the Care and Use of Laboratory Animals of the National Institutes of Health. Animal research protocols were also reviewed and consented to by the animal care committee of the Istituto Italiano di Tecnologia (permit 2007–2012). All surgical procedures were performed under anesthesia.

## Results

### Mouse Structural Connectivity Data

**Figure 1:**
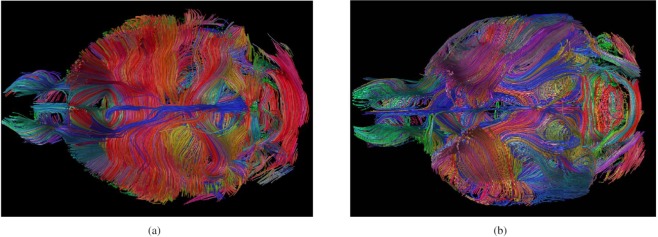
Example of the axial section tractography of (**a**) a normocallosal C57BL/6J control and an acallosal BTBR (**b**) mouse, where the different anatomical structures are apparent but difficult to understand. In particular, the lack of corpus callosum in (**b**) is visible.

To prove the discriminative power of our approach, we tested its ability to correctly distinguish the structural connectomes of two groups of mice (C57BL/6J and BTBR) characterized by previously described white matter alterations, i.e., the presence/absence of the two major neocortical intrahemispheric tracts, the corpus callosum and the dorsal hippocampal commissure^45^, as shown in Fig. 1. Because the structural alteration in the BTBR mice is well known, this dataset is used to validate the proposed method. Indeed, the BTBR mouse model represents a ground truth of expected differences between the two groups. Moreover, in addition to the discrimination between the groups, we are interested in empirically assessing the ability of our approach to correctly identify white matter tract differences in the two groups.

**Figure 2:**
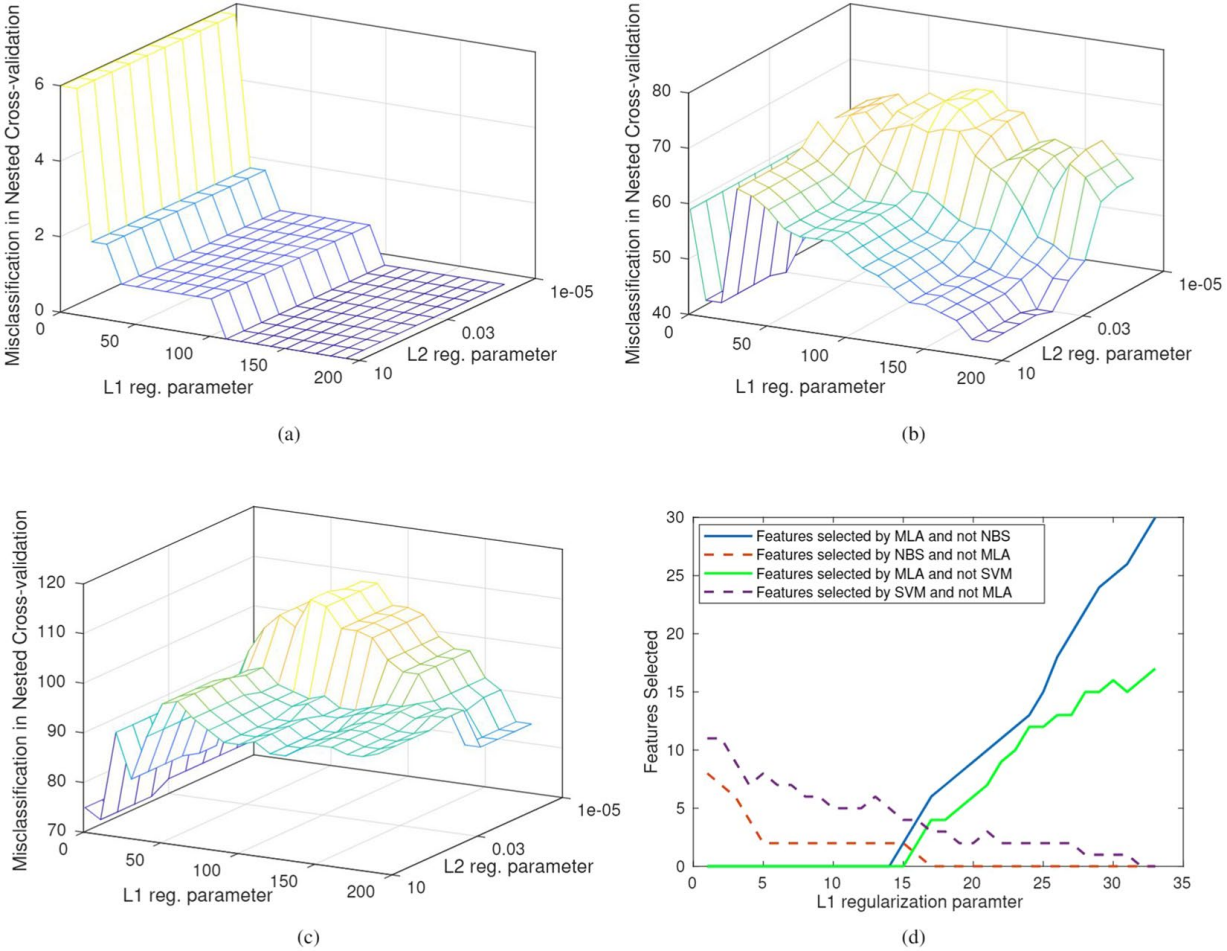
Misclassification error as a function of the regularization parameter computed with nested cross-validation. (**a**) Murine data experiment: The misclassification error reaches a plateau after *α* = 110, and the ${\ell }_{2}$ parameter *γ* has no influence. (**b**) Alzheimer human experiment: The misclassification has two plateaus, one near *α* = 20 and one near *α* = 190; for the ${\ell }_{2}$ parameter, *γ* > 0.03. (**c**) ADHD human experiment: The misclassification has a plateau near *α* = 10; the ${\ell }_{2}$ parameter changes the results but with minimal influence. (**d**) The number of features detected by one algorithm and not by the other varying the amount of sparseness. This graph shows that by decreasing the sparseness, the number of features detected by the MLA increases. The example shown is for the Alzheimer dataset.

Indeed, by using the proposed algorithm, the model correctly classified all samples in a cross-validation schema, and the structural differences—as the lack of corpus callosum—were found as expected from the literature. The mean misclassification resulting from the cross-validation when varying the parameter *α* is shown in Fig. 2(a).

**Figure 3:**
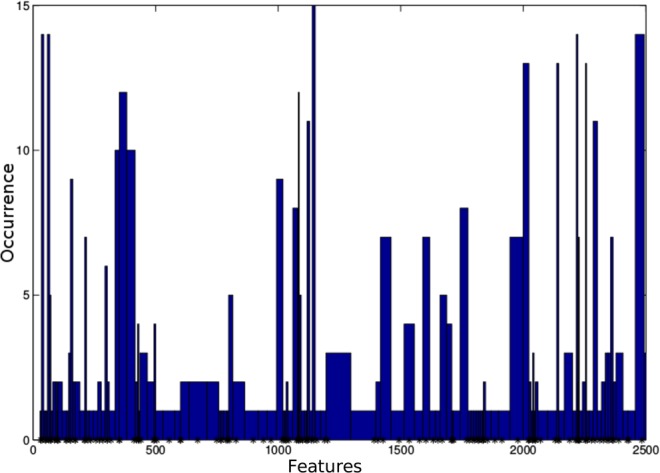
Murine data experiment: Histogram describing the occurrences of features (i.e., brain connections) selected in the mouse experiment. Higher values indicate connections that characterize the differences between the BTBR and control mice in the classifiers within our ensemble framework. This information is used to automatically select a subset of “relevant” features. Namely, the most frequent features highlighted by the histogram are retained.

**Figure 4:**
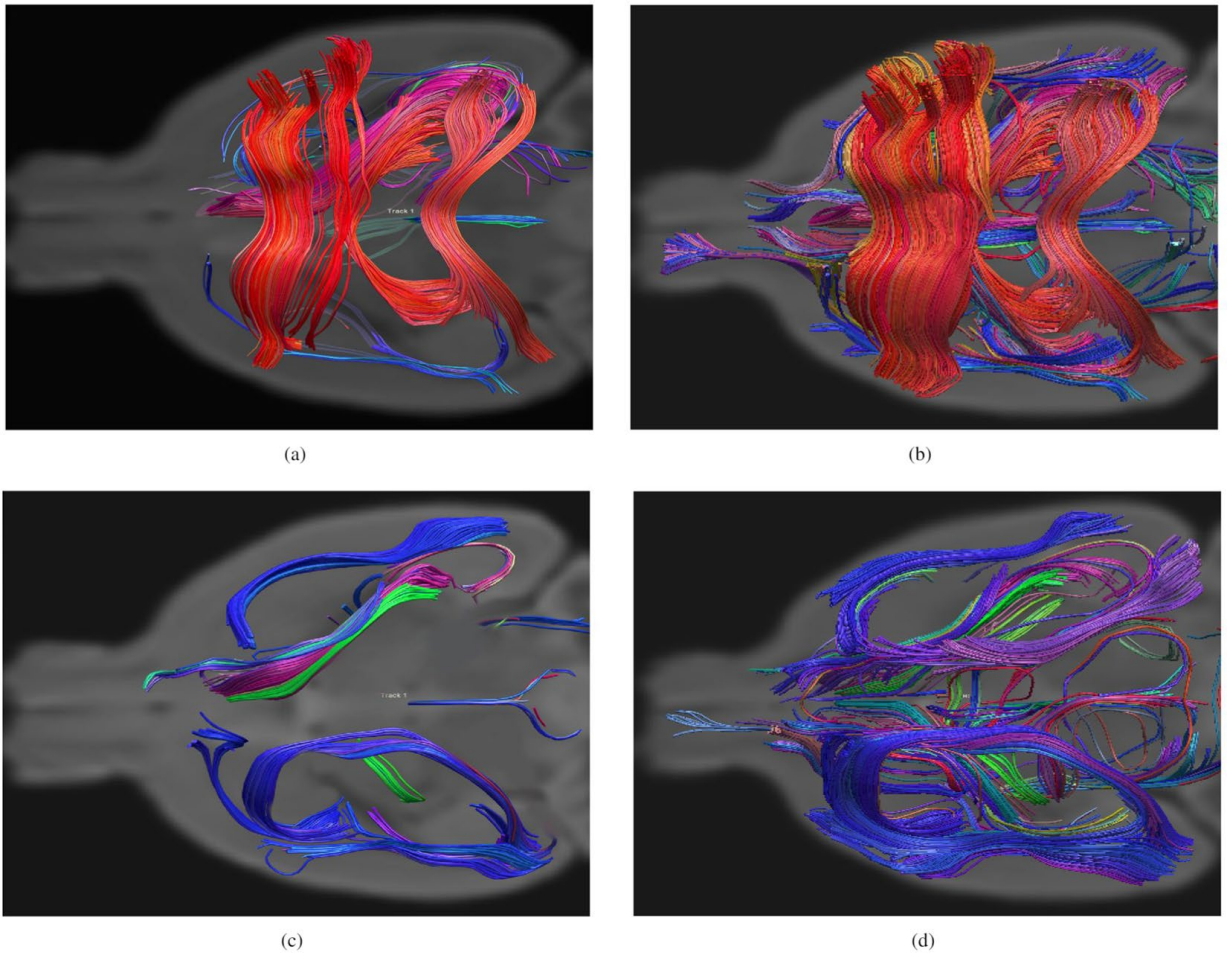
Graphical representation of the most significant features characterizing the structural connectome of the two populations: the axial views of a randomly selected subject from the C57BL/6J control population (**a**) using our algorithm (*α* = 110) and (**b**) the NBS algorithm using a threshold p-value of 0.05. (**c** and **d**) Are the axial views of a randomly selected subject from the BTBR population, respectively, for our algorithm and NBS. As expected, the BTBR mice show a lack of corpus callosum and hippocampal commissure and an increased intrahemispheric ipsilateral connectivity. Performing the same experiments by using the SVM framework, similar results to NBS were obtained. The depicted discriminant features detected by the proposed algorithm can be increased by varying the *α* parameter according to the user preference.

Thus, the proposed approach returns statistics about the relevance of the features by counting the number of occurrences of the features selected by the model ensemble. Figure 3 shows the occurrence of the detected features for the experiment with mouse structural connectomes, some of which are present in all the runs, indicating a strong relevance to the current research. Interestingly, the edges identified by the algorithm showed the expected characteristic features of the BTBR strain, including the agenesis of the corpus callosum and the presence of rostral-caudal rearrangement of white matter. Figure 4 shows how our algorithm (MLA) and NBS identify the parts of the corpus callosum that are known to be missing. The results obtained by using the SVM-based framework were also similar to those obtained by NBS. This experiment confirms that our new approach and NBS are able to identify the acallosal connections in the BTBR models.

Using MATLAB Mathworks 2014, the whole analysis, from the raw DTI data to the tract selection of the 16 subjects, took less than 40 minutes on a 2.6 GHz machine with 4 GB of RAM. However, the five rounds of MLA analysis required less than 1 second (with ${\ell }_{1}$ parameter *α* = 110 estimated by nested cross-validation, which also achieved 100% accuracy).

### Human Alzheimer Structural Data

We also tested the algorithm on a publicly available dataset based on human MRIs recorded from patients with Alzheimer’s disease compared with normal elderly subjects. The Alzheimer dataset was used to investigate the influence of the regularization parameter on the set of features selected by the proposed method compared to NBS (and SVM). In particular, we investigated the ability of the proposed approach to detect features that are not detected by NBS (or SVM) and vice-versa, varying the sparsity parameter *α*.

The selection of significant features with the NBS algorithm thresholded at p-value = 0.05 produced 14 connections. Using the detected features in a nested cross-validation case-control classification task produced an accuracy of 65%. The selection of significant SVM weights, instead, highlighted 20 connections, which in the nested cross-validation classification yielded 66% accuracy. It must be noted that some of the connections were detected by one algorithm and not by the other.

**Figure 5:**
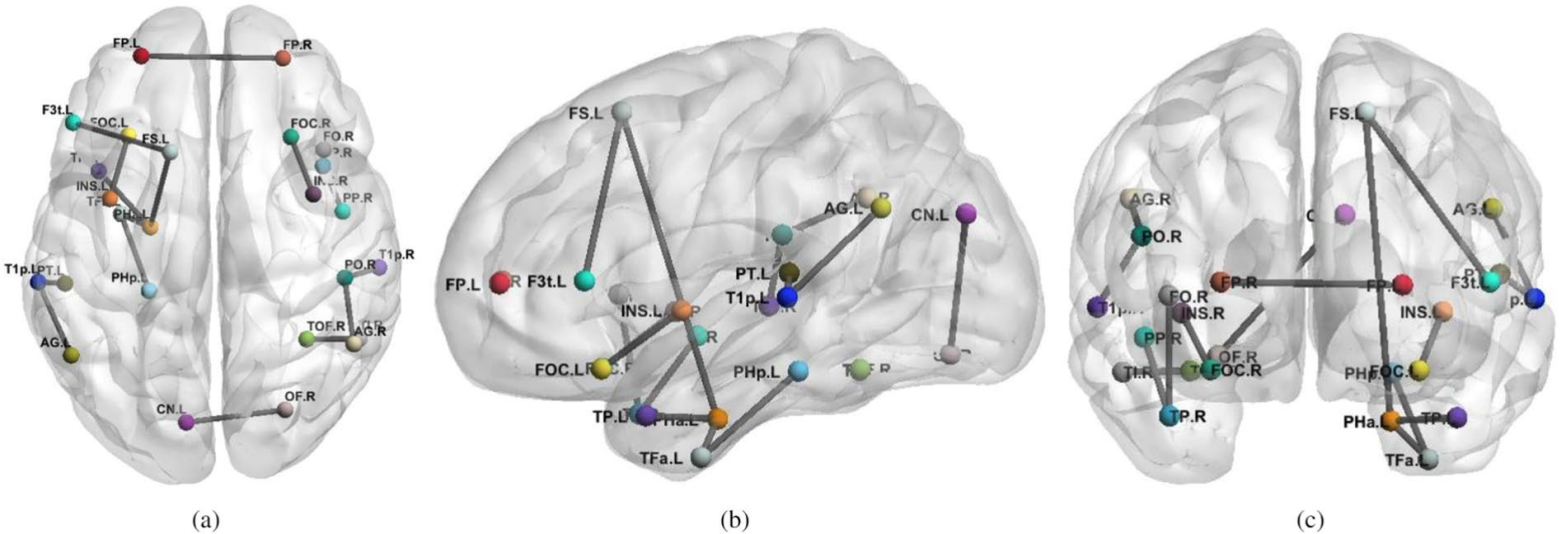
Structural connections differentiating patients with Alzheimer’s disease were obtained from elderly controls with MLA (our algorithm) using *α* = 33. From left to right, axial (**a**), sagittal (**b**), coronal (**c**) views of the brain indicate significant connections not set to zero by the algorithm. Each line represents a specific structural connection. The acronyms are the same as those reported in Table 1.

In contrast, with a proper choice of the sparsity parameter, the proposed approach detected all features selected by both NBS and SVM and some others. More specifically, as shown in Fig. 2(d), by decreasing the sparsity (i.e., increasing the parameter *α*), the number of features included by MLA and not by NBS (or SVM) increases, while the number of features detected as relevant by NBS (or SVM) and not by MLA decreases with a break-even point at *α* = 15. The best classification score with the features detected by the MLA was obtained with *α* = 33 with an accuracy of 75%. It is worthwhile to mention that generally detected features were symmetric. Namely, if a connection from ROI *a* to ROI *b* was detected, the reverse connection from *b* to *a* was also detected. The resulting features produced by the MLA approach are depicted in Fig. 5. The identified connections were mostly ipsilateral within the two temporal lobes. The analysis of the Alzheimer dataset took less than 1 second on a 2.6 GHz machine with 4 GB of RAM.

### Human ADHD Functional Data

By using the ADHD dataset, the cross-validation found the optimal solution for the MLA algorithm at *α* = 10, highlighting 8 discriminant connections across the groups with an accuracy of 70%. The NBS method, thresholded with a p-value = 0.05 did not find any significantly discriminative connections. The SVM-based framework, instead, showed 60% accuracy, detecting 2 significant connections.

The inability of NBS to find relevant connections might be because its first key step is the identification of candidate subnetworks, which are then tested for their relevance using a permutation test. These candidate subnetworks are selected only when the nodes are well connected; however, connectomes determined with high-dimensional parcellations (in our case, we used an atlas with 200 areas) are more likely to have a sparsely connected network. While this is a problem for methods expecting a densely connected graph, such as NBS, it does not affect our approach, which does not require any prior on the expected connectivity. The connections detected by the proposed algorithm are depicted in 2. The ADHD samples analysis took less than 30 seconds on a 2.6 GHz machine with 4 GB of RAM.

## Discussion

The proposed method performs a global multivariate analysis, characterizing local differences between networks. As this method is based on sparsity principles, it is particularly suited for those experiments with high-dimensional data and a small sample size. Moreover, the analysis based on multivariate statistics allows retrieval of subnetworks based on feature dependencies. The limitation of the NBS and the SVM-based approaches in detecting univariate differences is visible in the experiment with human functional data. In fact, the proposed algorithm detects some connections that are very often selected by the ensemble of learners, as seen in the histogram in Fig. 3. In contrast, with the univariate analysis, some edges are discarded as producing nonsignificant p-values. Nevertheless, the MLA, NBS and SVM approaches obtain similar results on the experiment with murine data, confirming the starting hypothesis on the anatomical differences between the two mouse lines.

The stability of the selected features is an important characteristic of the algorithm. As assessed empirically, increasing the value of *α* the model only introduces new features without removing features determined with smaller *α*s. The parameter *α* represents the strength and limitation of the method. In fact, although we determined the parameter automatically through cross-validation in the reported experiments, the sensitivity of the algorithm can be manually adjusted through this single parameter, which allows the neuroscientists to determine how strong the class characterization should be. A similar user-guided approach with methods based on sparsity has been previously described^34,35^.

When MLA was applied to the acallosal BTBR mice, a mouse model of autism^42,43^, as shown in Fig. 4, the tracts detected as discriminant were those with altered white matter connectivity in the BTBR mice with respect to the control mice. These results correspond with previous results in the literature, including the lack of corpus callosum and hippocampal commissure^46–48^ and increased intrahemispheric ipsilateral connectivity^49,50^, which were also observed in human patients with autism spectrum disorder (ASD)^51,52^. This result demonstrates that the algorithm is able to identify the known differences between groups.

Similar results were obtained with the structural dataset on Alzheimer’s disease. Altered brain connectivity both at the microstructural and macrocircuitry levels has been described in this disorder due to the amyloid plaques^53^. As depicted in Fig. 5, we found widespread temporal and parahippocampal connectivity differences in patients with Alzheimer’s disease compared to healthy elderly subjects. This finding is similar to several studies about functional connectivity that highlighted decreased functional connectivity between the temporal gyrus and neighboring regions^34,54^. Pathways between the hippocampus, the parahippocampal gyrus and the neocortical regions are considered to be the first affected in Alzheimer’s disease patients^55,56^. Neurodegeneration and loss of connectivity between frontal areas, the insula and within the areas of the frontal gyrus have also been documented^57^. The identified connection between the cuneal cortex left and the frontal operculum right was possible through fibers bifurcating from the corpus callosum. Studies have shown that the functional activities at the cuneal cortex are reduced with the progress of Alzheimer’s disease^58^; this can explain the visual field defects observed in some patients^59^.

Regarding the discriminant connections detected by the MLA algorithm for the ADHD dataset, among the detected areas using *α* = 10, there were connections between the frontal pole and cingulate gyrus,and the frontal pole and angular gyrus, which are the main functional hubs of the default mode network (DMN). The DMN is known to be altered in ADHD subjects^60,61^, as it has been hypothesized that ADHD subjects may have a diminished ability to inhibit the default processing of the DMN^62^. The other detected connections could be explained as dorsal medial and medial temporal systems related to the DMN^63^.

**Figure 6:**
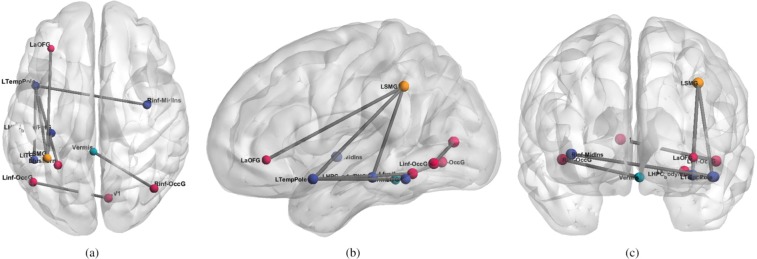
Functional connections differentiating ADHD from TD subjects by using the proposed method (MLA) using *α* = 10. From left to right, axial (**a**), sagittal (**b**), coronal (**c**) views of the brain indicate significant connections not set to zero by the algorithm. Each line represents a specific functional connection. For details on the statistics and name abbreviations, see Table 2.

The connectivity differences for the human dataset, shown in Figs 5 and 6 and reported in Tables 1 and 2, were those detected by MLA, some of which were not discovered by NBS and SVM. This result shows that different approaches—one based on sparsity and one based on a familywise error rate—can produce similar results, though the proposed method finds more features than the NBS or other univariate approaches. NBS and MLA select discriminative features in different ways. NBS performs univariate t-tests among the features, while MLA performs a sparse multivariate regression. It is acknowledged that the methods achieve different objectives. However, it has been shown that a sparse model combined with stability selection criteria can lead to more robust results than models based on false discovery rate or familywise error-rate^64^. The advantage of MLA resides in the possibility of using it in an exploratory framework, where it provides insights (detected connections) to neuroscientists for a deeper investigation.

**Table 1:** Structural connections differentiating patients with Alzheimer’s disease from normal elderly individuals detected by MLA.

#	Region 1	Region 2	p-value	
			NBS	SVM
1	Insula-L (INS.L)	Frontal Orbital Cortex-L (FOC.L)	0.0002	<0.05
2	Insula-L (INS.L)	Inferior Frontal Gyrus, pars opercularis-L (F3t.L)	0.0002	N.D.
3	Superior Frontal Gyrus-L (FS.L)	Inferior Frontal Gyrus, pars opercularis-L (F3t.L)	N.D.	N.D.
4	Superior Frontal Gyrus-L (FS.L)	Parahippocampal Gyrus, ant. div.-L (PHa.L)	0.01	N.D.
5	Temporal Pole-L (TP.L)	Parahippocampal Gyrus, ant. div.-L (PHa.L)	0.0003	<0.05
6	Temporal Pole-R (TP.R)	Frontal-Operculum-R (FO.R)	N.D.	<0.05
7	Temporal Pole-R (TP.R)	Planum Polare-R (PP.R)	0.0003	<0.05
8	Superior Temporal Gyrus, post. div.-L (T1p.L)	Angular Gyrus-L (AG.L)	N.D.	N.D.
9	Superior Temporal Gyrus, post. div.-L (T1p.L)	Planum Temporale-L (PT.L)	0.001	<0.05
10	Superior Temporal Gyrus, post. div.-R (T1p.R)	Parietal Operculum-R (PO.R)	0.0003	<0.05
11	Inferior Temporal Gyrus, temporooccipital-R (TO3.R)	Temporal Occipital Fusiform-R (TOF.R)	N.D.	<0.05
12	Angular Gyrus-R (AG.R)	Parietal Operculum-R (PO.R)	N.D.	N.D.
13	Cuneal Cortex-L (CN.L)	Frontal Operculum-R (OF.R)	N.D.	<0.05
14	Insula-R (INS.R)	Frontal Orbital Cortex-R (FOC.R)	0.006	N.D.
15	Parahippocampal Gyrus, ant. div.-L (PHa.L)	Temporal Fusiform Cortex, ant. div.-L (TFa.L)	0.001	N.D.
16	Temporal Fusiform Cortex, ant. div. (TFa.L)	Parahippocampal Gyrus, post. div.-L (PHp.L)	0.01	<0.05
17	Temporal Pole-L (TP.L)	Temporal Pole-R (TP.R)	N.D.	<0.05

Pairs of source and target regions and p-values of the univariate t-test computed on NBS^26^, and SVM weights using the t-test threshold corresponding to p-values < 0.05^20,21^ are reported. “Not detected” (N.D.) means that there is no significant difference between the two areas.

**Table 2:** Functional connections differentiating patients with ADHD from TD individuals.

#	Region 1	Region 2	p-value	
			NBS	SVM
1	Temporal Pole-L	Inferior Temporal Gyrus-posterior-division-L	N.D.	N.D.
2	Temporal Fusiform Cortex anterior division-L	Temporal Pole-L	N.D.	N.D.
3	Frontal Orbital Cortex-L	Supramarginal Gyrus posterior division-L	N.D.	N.D.
4	Temporal Pole-L	Supramarginal Gyrus posterior division-L	N.D.	N.D.
5	Supramarginal Gyrus posterior division-L	Parahippocampal Gyrus anterior division-L	N.D.	N.D.
6	Cerebellum Vermis VI	Inferior Occipital Cortex-R	N.D.	<0.05
7	Middle Temporal Gyrus anterior division-R	Lateral Occipital Cortex inferior division-L	N.D.	<0.05
8	Temporal Pole-L	Inferior Insular Cortex-R	N.D.	<0.05

Pairs of source and target regions and p-values of the univariate t-test computed on the NBS^26^ and SVM weights^20,21^ are reported. “Not detected” (N.D.) means that there is no significant difference between the two areas. ADHD = attention-deficit/hyperactivity disorder, TD = typically developed. For this experiment, no statistically significant features were obtained by the NBS and SVM-based algorithm using the t-test threshold corresponding to p-value < 0.05^20,21^.

## Conclusions

In this manuscript, a fully automated method to characterize brain connectivity in case-control studies was reported. The method, based on a sparse learning classification, was tested on structural and functional connectivity data. The approach is able to identify brain areas of interest that can be further analyzed with standard seed-based approaches or through histological white matter validation.

The algorithm successfully highlighted some known structural white matter differences in acallosal mice and identified previously reported alterations of structural and functional connections in human Alzheimer’s and ADHD patients. The developed software is freely distributed as a MATLAB toolbox at the url https://github.com/alecrimi/multi-link. Our approach can help to highlight the differences in connectivity generating hypotheses that can complement univariate techniques.

## Methods and Data

This section first describes the two types of data used to test the proposed method: a mice dataset with high dimensionality, and two publicly available human datasets. Afterwards, the preprocessing and the proposed computational model for discriminating patterns in the whole-brain analysis are described.

### Data

#### Mouse Structural Connectivity Data

The mouse cohort was composed of two groups of 22- to 26-week-old male subjects (n = 16): BTBR T+ tf/J mice (n = 8), which share analogies to all diagnostic symptoms of autism and characteristic functional and structural features of the brain^47–49^, and C57BL/6J mice (n = 8), which are characterized by normal sociability and represent the control group. Figure 1 depicts an example of the expected difference between the BTBR and C57BL/6J mouse groups. In particular, BTBR mice lack the corpus callosum, which is different from the C57BL/6J mice.

The animal preparation protocol has been previously described^45,49^. Briefly, brains were imaged inside intact skulls to avoid postextraction deformations. *Ex vivo* high-resolution DTI and T2-weighted images were acquired on paraformaldehyde-fixed specimens with a 7 Tesla Bruker Pharmascan MRI scanner (Billerica, MA, USA). T2-weighted MR anatomical images were acquired using a RARE sequence with the following imaging parameters: TR/TE = 550/33 ms, RARE factor = 8, echo spacing 11 ms, and a voxel size of 90 *μm* isotropic. DTI volumes were acquired using 4 scans at b0 and 81 scans with different gradient directions (b = 1262 s/mm^2^), with resolution 130 × 130 *μm*^2^, using a 4-shot EPI sequence with TR/TE = 5500/26 ms. Anatomical and DTI sequences were acquired sequentially at the same center with the same scanner. This dataset is freely distributed^65^. This dataset is used to show that the algorithm is able to identify the difference between the groups, which are expected to be found as a proof of concept.

#### Human Structural Connectivity Alzheimer Data

The human experiments were performed on the Alzheimer’s Disease Neuroimaging Initiative (ADNI) dataset, which is publicly available^66^. Only baseline scans were used to avoid confounding factors as advanced brain atrophy and treatment in the Alzheimer patients. This cohort comprised 51 Alzheimer’s disease patients (age: 76.5 ± 7.4 years), and 49 normal elderly subjects (77.0 ± 5.1) matched by age. The data used were DTI, and T1-weighted obtained by using a GE Signa scanner 3 T (General Electric, Milwaukee, WI, USA). The T1-weighted scans were acquired with voxel size = 1.2 × 1.0 × 1.0 *mm*^3^ TR = 6.984 ms; TE = 2.848 ms; flip angle = 11°). DTI were acquired at voxel size = 1.4 × 1.4 × 2.7 *mm*^3^, scan time = 9 min, and 46 volumes (5 T2-weighted images with no diffusion sensitization b0 and 41 diffusion-weighted images b = 1000 *s*/*mm*^2^). For each subject, DTI and T1 were acquired and coregistered.

#### Human Functional Connectivity ADHD Data

Functional connectivity was also investigated on a larger resting-state fMRI dataset comprising ADHD and TD subjects^67^. In particular, we used the publicly available New York University Child Center dataset^68^, which is the main cohort of this study. The dataset comprised 95 ADHD subjects (67 male and 28 female, mean age 11.4 ± 2.7), who were either inattentive, hyperactive or both, and 92 healthy TD subjects (45 male and 47 female, mean age 12.4 ± 3.1), who represent the control group.

The fMRI volumes were acquired with a Siemens Allegra 3 T, with TR/TE 2000/15 ms and voxel size 3 × 3 × 3 *mm*^3^. This dataset is used as a real case study where NBS and other univariate approaches are not useful in proving local connectivity differences.

### Methods

#### Mouse Dataset Processing and Encoding

Deterministic tractography was performed on the DTI volumes by using the software tool DiPy^69^ after eddy current corrections, using the fiber assignment by continuous tracking (FACT) algorithm^70^. Fibers were reconstructed in the original volumes following the 2nd-order Runge-Kutta integration scheme^71^ starting from the center of each voxel and following the main direction of the tensor. The tracking was stopped when the fiber made a sharp turn (>35°) or entered a voxel with fractional anisotropy (FA) < 0.15.

To allow intersubject comparisons, registration matrices to a common space were computed for each subject by using affine transformation (12 degrees of freedom). The obtained registration matrices were then applied to the endpoints of each fiber. This approach allowed the tractography algorithm to work on the original volume space without warping the tensors.

To enable purely data-driven intergroup comparisons without the use of anatomical priors, the brain volumes were split into 3D cubes of size 1 × 1 × 1 *mm*^3^, without considering any atlas. Each cube was a node in the graph, and the connectivity matrix was built counting the fibers starting and ending in two distinct cube elements of the grid, avoiding the inclusion of u-fibers. This process resulted in defining 42,704 edges.

The advantage of this approach was that the result of the proposed analysis method was nearly independent of the size and the type of parcellation. Indeed, not considering the anatomy nor the physiology of the brain might result in bundles of fibers split into “subbundles” connecting adjacent cubes. However, if there is a difference between the two groups, it is retrieved for all subbundles, and hence, the overall bundles are then reconstructed. However, the choice of using a fine grid or an atlas is arbitrary.

#### Alzheimer Dataset Processing and Encoding

Tractographies for all subjects were generated by processing the DTI data with a deterministic Euler approach of DiPy^69^, stemming from 2,000,000 seed-points and stopping when the FA was smaller than 0.25. Tracts shorter than 3 cm were discarded during the connectome construction. Structural connectivity matrices were constructed by counting the number of fibers connecting two regions of interest (ROIs) of the registered Harvard-Oxford atlas^72^. This atlas defines 96 ROIs; it is freely available with several brain imaging analysis platforms, and it has been used in several structural studies including Alzheimer’s disease^73^. The Harvard-Oxford atlas is a probabilistic atlas. However, we used the version where each voxel is associated with the ROI with the highest probability. The choice of different algorithms used for the tractography with the human and murine data is related to the fact that the data are obtained with different types of scanners: a small animal device and a common clinical scanner.

#### ADHD Dataset Preprocessing and Encoding

This dataset was preprocessed^60^, and the final connectivity matrices are publicly available^68^. In brief, resting-state fMRI data were preprocessed following these steps: Removal of first 4 EPI volumes, slice timing correction, and motion correction, and then, application of the regressors for WM, CSF, motion time courses and a low order polynomial detrending. A bandpass filter of 0.009 < *f* < 0.08 Hz was also applied. Finally, the data were blurred using a 6-mm full width at half maximum Gaussian filter. The functional region of interests was obtained using the Craddock parcellation^74^ for 200 areas. These preprocessing steps were carried out according to the Athena pipeline^75^, which is based on a combination of commands from AFNI and FSL.

### Parameter Tuning

While the number of discriminative connections selected by our type of model is tuned by the choice of *η*, we noticed that the algorithm was satisfactorily discriminating the two classes on a wide range of *η* values. In this work, we were mostly interested in discriminant features rather than finding an optimal classification. However, the results are shown using the values which allow better accuracy estimation in a nested cross-validation manner to produce a jackknife-like classification. In practice, a nested leave-one-out procedure was employed. For 1000 iterations, a sample was removed from the training dataset and used as the test-set to find the optimal value within a range, and then, the performances were evaluated on another sample that was also removed prior to the optimization from the training set and used as validation. In the presence of a plateau of identical optimal values, the value generating fewer connections was taken. The range of values was previously identified empirically. Namely, several values were tried to identify those showing meaningful connectivity.

The optimal parameter *γ* was investigated in a grid search for all experiments. It was noted that, in contrast to the parameters of the other models, the value of this parameter was not critical for the classification. Nevertheless, values larger than 0.03 were producing slightly better results than smaller values.

Although this model can be very powerful in determining a small and good subset of features allowing the linear discrimination of classes, it suffers from a stability problem^28^, i.e., small changes in the data could change the result of a single run. To address this stability issue to improve the robustness of SDA, we introduced a second stage, exploiting the ensemble of low-stability algorithms to produce a more stable feature selection. In practice, we performed further feature selection to ensure that the features are stable across subjects.

In the specific case, the SDA classifier was trained with a nested leave-one-out approach. This ended in an ensemble of models, each one with a subset of “relevant” features (connections) selected to maximize the discrimination between the two groups. Then, we refined this ensemble of models by occurrence validation, where only features which were frequently selected during cross-validation were retained, i.e., features occurring in less than a predefined percentage of runs were discarded.

In all the experiments reported in the paper, this threshold was determined as half the number of subjects in the corresponding dataset. Thus, we ensure a stable selection of the features^28^. The choice of using half the number of subjects as the threshold for sampling features was used previously^64,76^ as a trade-off between considering all features (no restriction) and considering only features which occur in all samples (overrestrictive). The impact of varying the threshold was investigated previously^64^, showing that a small number of selected features (obtained by thresholding) guarantees a small number of false positives. Nevertheless, the focus of the paper is on obtaining an optimal true positive rate rather than a low number of false positives.

## Supplementary information

Appendix
