# Supplementary material for: MultiLink Analysis: Brain Network Comparison via Sparse Connectivity Analysis
Source: Sci Rep. 2019 Jan 11;9:65. doi: 10.1038/s41598-018-37300-4 (PMC6329758; doi:10.1038/s41598-018-37300-4)
Supplement: Supplementary file 1 — Appendix [file 41598_2018_37300_MOESM1_ESM.pdf]

MultiLink Analysis: Brain Network Comparison via Sparse Connectivity Analysis
Alessandro Crimi, Luca Giancardo, Fabio Sambataro, Alessandro Gozzi, Vittorio Murino, and Diego Sona

Appendix

Sparse Discriminant Analysis

The general formulation of the ℓ_1 regularization or *lasso* is used in regression frameworks to minimize the problem $min_\beta\{||\mathbf{y}-\mathbf{X}\beta||^2+\eta||\beta||_1\}$, where $\mathbf{X}$ is a data matrix, $\mathbf{y}$ is the output vector, and β is the regressor vector. Similarly, the elastic net is given as $min_\beta\{||\mathbf{y}-\mathbf{X}\beta||^2+\eta||\beta||_1+\gamma||\beta||_2\}$. In these equations, η and γ are tuning parameters that are used to yield sparse coefficient vector estimation[41]. The parameter η can also be reformulated as the number of desired variables that are left in the model; when used in this context we refer to it as α[41].

There are several extension to the linear discriminant analysis[78] which comprises Lasso and elastic net[40]. Our experiments are based on the formulation proposed by Clemmensen et al[30]. More specifically, given the matrix data $\mathbf{X}$ with n p-dimensional observations for K=2 classes, each of them defined as $\mathbf{x_i}$, with $\mu_\mathbf{k}$ representing the mean for a specific class k, it is possible to define the within-class covariance matrix common to all classes as $\Sigma_w=\frac{1}{n}\sum_{k=1}^{K}\sum_i(\mathbf{x_i}-\mu_\mathbf{k})(\mathbf{x_i}-\mu_\mathbf{k})^T$, and the between-class covariance matrix $\Sigma_b=\sum_{k=1}^{K}\pi_k\mu_\mathbf{k}\mu_\mathbf{k}{}^T$, where π_k is the prior probability for each class to belong to the class k. The prior probability is generally given by the size of the respective classes.

A Fischer discriminant analysis can classify to which class a sample belongs by using discriminant vectors whose directions $\beta_\mathbf{k}$ maximize

$$\max_{\beta_\mathbf{k}}\beta_\mathbf{k}^\mathbf{T}\Sigma_b\beta_\mathbf{k}, \tag{A.2}$$

subject to $\beta_\mathbf{k}^\mathbf{T}\Sigma_w\beta_\mathbf{k}=1$ and $\beta_\mathbf{k}^\mathbf{T}\Sigma_w\beta_\mathbf{l}=0\ \forall l<k$.

Very often, as in our case, the previous maximization process is ill-posed, as the matrix Σ_w might not be full rank as the number of features is far larger than the number of available samples. A possible solution, proposed by Witten et al.[40], is given by using the Lasso or elastic net regularization as

$$\max_{\beta_\mathbf{k}}(\beta_\mathbf{k}^\mathbf{T}\Sigma_b\beta_\mathbf{k}-\eta||\beta_k||_1-\gamma||\beta_k||_2) \tag{A.3}$$

subject to $\beta_\mathbf{k}^\mathbf{T}\Sigma_w\beta_\mathbf{k}=1$ and $\beta_\mathbf{k}^\mathbf{T}\Sigma_w\beta_\mathbf{l}=0\forall l<k$. Alternatively, we used the minimization formulation of Clemmensen et al.[30], where the pair given by $\beta_\mathbf{k}$ and the vector of scores $\theta_\mathbf{k}$ solves the problem

$$\begin{cases} min_{(\beta_k,\theta_k)}||\mathbf{Y}\theta_\mathbf{k}-\mathbf{X}\beta_\mathbf{k}||^2+\eta\ ||\beta_k||_1+\gamma\ \beta_k^T\Omega\beta_k, \\ \text{subject to } \frac{1}{n}\theta_\mathbf{k}^\mathbf{T}\mathbf{Y}^\mathbf{T}\mathbf{Y}\theta_\mathbf{k}=1,\theta_\mathbf{k}^\mathbf{T}\mathbf{Y}^\mathbf{T}\mathbf{Y}\theta_\mathbf{l}=0\ \forall l<k, \end{cases} \tag{A.4}$$

where Ω is an arbitrary positive matrix, η and γ are nonnegative tuning parameters, and $\mathbf{Y}$ is an $n\times K$ matrix of dummy variables for the K classes. This formulation of LDA as a regression problem introduces sparsity and allows its use when the number of features is very large compared to the number of available samples.
